# Emergency vaccination of rabies under limited resources – combating or containing?

**DOI:** 10.1186/1471-2334-5-10

**Published:** 2005-03-07

**Authors:** Dirk Eisinger, Hans-Hermann Thulke, Thomas Selhorst, Thomas Müller

**Affiliations:** 1 Department of Ecological Modelling, UFZ-Centre for Environmental Research Leipzig/Halle, Leipzig, Germany; 2 Friedrich-Loeffler-Institut, Federal Research Institute for Animal Health, Wusterhausen, Germany

## Abstract

**Background:**

Rabies is the most important viral zoonosis from a global perspective. Worldwide efforts to combat the disease by oral vaccination of reservoirs have managed to eradicate wildlife rabies in large areas of central Europe and North-America. Thus, repeated vaccination has been discontinued recently on a geographical scale. However, as rabies has not yet been eradicated globally, a serious risk of re-introduction remains. What is the best spatial design for an emergency vaccination program – particularly if resources are limited? Either, we treat a circular area around the detected case and run the risk of infected hosts leaving the limited control area, because a sufficient immunisation level has not yet been built up. Or, initially concentrate the SAME resources in order to establish a protective ring which is more distant from the infected local area, and which then holds out against the challenge of the approaching epidemic.

**Methods:**

We developed a simulation model to contrast the two strategies for emergency vaccination. The spatial-explicit model is based on fox group home-ranges, which facilitates the simulation of rabies spread to larger areas relevant to management. We used individual-based fox groups to follow up the effects of vaccination in a detailed manner. Thus, regionally – bait distribution orientates itself to standard schemes of oral immunisation programs and locally – baits are assigned to individual foxes.

**Results:**

Surprisingly, putting the controlled area ring-like around the outbreak does not outperform the circular area of the same size centred on the outbreak. Only during the very first baitings, does the ring area result in fewer breakouts. But then as rabies is eliminated within the circle area, the respective ring area fails, due to the non-controlled inner part.

We attempt to take advantage of the initially fewer breakouts beyond the ring when applying a mixed strategy. Therefore, after a certain number of baitings, the area under control was increased for both strategies towards the same larger circular area. The circle-circle strategy still outperforms the ring-circle strategy and analysis of the spatial-temporal disease spread reveals why: improving control efficacy by means of a mixed strategy is impossible in the field, due to the build-up time of population immunity.

**Conclusion:**

For practical emergency management of a new outbreak of rabies, the ring-like application of oral vaccination is not a favourable strategy at all. Even if initial resources are substantially low and there is a serious risk of rabies cases outside the limited control area, our results suggest circular application instead of ring vaccination.

## Background

Rabies is life-threatening for humans [1] and the most important viral zoonosis from a global perspective [2]. In Europe and North-America, wildlife is the main reserve (i.e. foxes or raccoons). Aerial distribution of vaccine filled baits proved to be a method which can be used for controlling rabies in these species, as they are attainable via baits, and an efficient oral rabies virus vaccine is available [3,4]. Therefore, disease managers have been making huge efforts in rabies control over the last 25 years [2,5-9]. Long-term and large scale oral vaccination of wildlife eradicated rabies at the regional scale in central Europe and the Americas [10-16]. Consequently, repeated vaccination in these regions has now ended [8,16,17] and, eventually, its host populations will be completely susceptible to new rabies infection. Therefore, we must be aware of a reintroduction as long as rabies persists anywhere in the world, [18] and we have to develop emergency measures designed for a local outbreak in non-immunised wildlife populations. Thus recent contingency planning appears comparable to the situation in the UK at the end of the last century when an introduction from continental Europe was expected [19-23]. A lot of literature is available from that period concerning how a newly introduced rabies epidemic potentially spreads or will be controlled [21,22,24,25]. However, empirical knowledge has been accumulated in the mean time regarding large-scale field application of oral vaccination, recognition of successful strategies or operating population immunity levels, and termination of repeated baiting. It appears worthwhile to exploit these sources, in order to adjust contingency plans for future rabies control in general and the event of rabies re-introduction in particular.

How should disease management react to re-introduction, i.e. detection of an infection within a rabies-freed area? Revitalising country-wide vaccination campaigns appears to be not very well-adapted to detection of a local rabies outbreak [26]. A WHO [27] recommendation suggests 5,000 sq. km of compact vaccination area as the minimum sustainable strategy, but there are no details regarding the plausible spatial configuration. Field practice demonstrates that modern aerial distribution of vaccine-filled baits performs precisely, even on complex spatial distribution patterns [28,29].

Thus, alternative control application schemes can be considered as emergency strategies, which 1) are able to restrict the spatial extent of the control area, 2) are able to eradicate the disease and finally, 3) are logistically practicable.

Disease managers usually think of combating an outbreak by immediately controlling all areas at risk [17]. But in an emergency, when the outbreak is very local at first, to what spatial extent must a control area be designed to cover "all areas at risk", or in practice, to what distance might the disease spread until a protective immunisation level has been built up [24,26,30]? How can we exclude potential breakouts of infected hosts just before the control measures succeed in the controlled area? The most appealing counter-measure would fence in the epidemic first and eradicate afterwards. The "fence" could be realised by a ring-shaped area of competently vaccinated hosts at an adequate distance from the detected outbreak (see for example [31]). The host population of such a ring area is already well immunised before first infections will reach the inner border of the ring – hence the outbreak is actually contained. But, the ring approach promises another advantage: Although we BAIT an equally sized area compared to circle application, the larger spatial extent of the ring allows for an increased control area because the outer border of the ring is beyond the circle of respective size. Indeed, the inner part of the ring must not be treated in the beginning which could be important if we have to cope with logistic and/or resource limitations immediately after an outbreak (Vos, pers. comm). While aiming at a serious contingency plan, we still have to analyse the comparability of the two different approaches from the epidemiological discussion: Centred on the detected outbreak, we treat either a circular compact area or the equal-sized area, but arrange it in a ring around an omitted inner part (i.e. equal size of baited area, equal baiting program, equal number of baits and same bait density).

**Figure 1:**
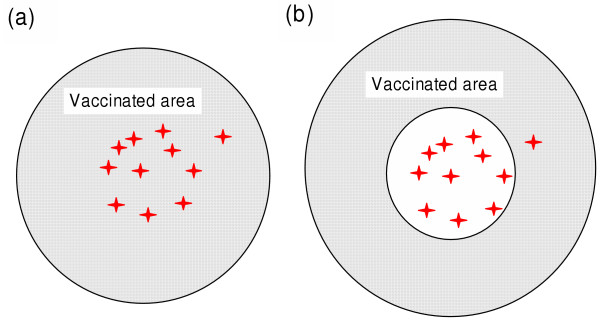
**Spatial design of an emergency vaccination**. Schematic design of the vaccinated area in an emergency situation (hashed – vaccinated area, blank – not vaccinated, stars – detected cases of rabies). (a) Circle design: The surrounding area of the first detected case of rabies is vaccinated. (b) Ring design: The immediate surroundings are not vaccinated, but a ring-shaped surface around the detection area is vaccinated.

We have identified two strategic alternatives: (i) *Combating *– refers to vaccination applied in a *circle *which (a) aims at immediate treatment of the surrounding area of the outbreak to keep the number of rabies cases low, but (b) accepts the risk of early breakout due to an unfinished build up of immunisation level. (ii) *Containing *– refers to *ring *vaccinations applied at a distance from the outbreak which (a) aims to prevent a breakout of rabid animals through a readymade immunisation level within the ring, before the epidemic reaches it, but (b) accepts higher numbers of cases in the inner part (Fig. 1).

We use an explicit simulation model of the fox-rabies system to compare the different spatial designs of vaccination. We analyse how long the circle or the ring design can keep the rabies epidemic inside the control area. We compare the two spatial designs for the application of mixed strategies, i.e. the definition of most-rewarding-point-in- time, in order to change from ring to circle, as compared to a pure circle strategy. In case of a rabies outbreak in a previously rabies-free region, the results determine which of the strategies should be applied and how to benefit the most from limited resources.

## Methods

### Model background

We evaluated the management strategies with a model of the rabies-fox system which is tailored to emergency control planning. We applied a spatially-explicit, individual-based, time-discrete modelling approach [32-34]. This approach had already proved practical in studying the spread and control of rabies in foxes [26,35-37] and to provide useful management support [17]. Thus, previous models [26,35,36] were enhanced in order to cope with the new question. The rules of rabies dynamics between the fox family groups, as well as individual dispersal of juveniles after maturity, were adopted from the basic model [35,36]. The spatial unit of a fox group home-range was also maintained because it had proven suitable for studying the disease spread on the regional scale [20,38,39]. Group home-ranges are implemented within regular grid cells, and the obtained results would not change if the grid cells were replaced by irregular shaped home-ranges of a given mean-size. This is because the dispersal movements are modelled relative to group home-range size [35,40,41] and not in metric measures [36,42-45]. This approach incorporates an implicit adjustment of the fox density effect [40]. A metric reference to fox densities within central Europe is realised by means of the mean-fox-group home-ranges (i.e. cells) corresponding to 1 sq. km[46]. Compared to previous rabies models which had addressed an introduction of rabies [20,25,31,47,48], it was necessary to extend the simulation area (i.e. 256 times 256 cells corresponding to ~65.536 sq. km in a central European scenario) in order to allow relevant dimensions of the control area. The representation of the control area by a regular ring or circle within the model is an abstraction. When applied to real landscapes the vaccination areas are non-regular, as they are usually determined by administrative borders, hence certain excess areas must be baited additionally. Thus the geometric simplification in the model represents the required core area, which must at least be covered by the vaccination area defined along administrative units. But, the aim of our study necessitates a further step in scaling down the basic model [49]. The temporal resolution was refined to a weekly time step, since the success of an emergency vaccination depends on the time of introduction of rabies into the fox population, the time until detection of the epidemic, and the timing of the initial vaccination campaign [7,17,20]. The model rules were complemented with ecological characteristics of, and disease transmission between, individual foxes of a group. The individual-based representation enables a locally varying immunisation level due to non-homogeneous bait uptake [50,51] or individual foxes moving across the border of the vaccination area. The effect of these issues might be negligible for vaccination success on a geographical scale, [36] but it becomes serious for the few rabid animals after an outbreak or a spatially limited vaccination area WITHIN a landscape.

### Basic fox population model

Each cell comprises a family group [38,52] of age-classified individual foxes (juvenile, adult). Fox groups in the field contain on average 2–3 adults (i.e. 1 male and 1–2 females) before reproduction [52-55]. The pattern is realised in the model by assuming a maximum group size of 5 adults [46,56] together with the mortality and dispersal process. For parameterisation see Table 1.

**Table 1:** Parameters of the model, default values and reference.

**Parameter**	**Value**	**Reference**
**Population Ecology**		
PMaxAdultsPerGroup	5	Adjusted to [46]
PMonthlyMortalityAdults	6.1 %	[58]
PMonthlyMortalityJuveniles	12.0 %	[58]
PLitterMeanSize	5.5	[44,64,117]
PLitterStdDev	1.5	[44]
**Juveniles' Dispersal**		
PDispersalProbabilityNotToLeave	15.0 %	[43,44,70]
PDispersalIntrinsicMaxDistance	60 steps	[35]
PDispersalMaximumDistance	100 steps	[43,70]
PDispersalMortalityPerStep	2.0 %	Adjusted to [69]
PDispersalLengthOfDispersalPeriod	8 weeks	[67]
**Rabies Epidemiology**		
PIncubationPeriodMean	3.5 weeks	[74]
PIncubationPeriodMinimum	2 weeks	[74]
PTransmissionProbabilityPerNeighbourGroup	14.0 %	Following [36,76]
PTransmissionBasicProbabilityMaiting	14.0 %	Following [36,76]
**Management Strategy**		
PManagementDetectionProbablity	2.0 %	[24,93]
PManagementBaitDensity	20 bpkm^2^	[7,10,11,13,17,79]
PVacArea	6,400 10,800 16,000 cells	**Variable in accordance with simulation experiments**

#### Mortality

Without rabies, adult foxes have a monthly mortality of 6.1% [57,58]. Juveniles are subjected to a monthly mortality of 12% until dispersal [58]. After dispersal they are treated as adults [59-61].

#### Reproduction

Reproduction is scheduled in the first week of April. All non-empty cells produce a litter of a normally distributed number of cubs with mean of 5.5 and a standard deviation of 1.5 [44,57,58,62-64]. Fox groups which consist of exactly one individual reproduce with 50% probability. This rule accounts for floaters and multiple mating males as well as for non-reproducing males [53,65,66].

#### Dispersal

With these population dynamics, on average 3.5 juveniles per group emerge in the maturity dispersal (Goretzki, pers. comm.). The dispersal occurs for 8 weeks from October to November [64,67]. Thus, during that phase per time-step one eighth of all cells are selected randomly. Out of each selected cell, all juveniles move consecutively according to the following dispersal algorithm. The dispersing individual is randomly assigned with a main direction from 8 cells of 360 degrees, which is maintained in each step with 50% probability [60,67-69]. In the remaining steps the individual deviates to the left or to the right by one cell with equal probability (i.e. 25%; see [35]). The probability to settle in a cell (PSettle) increases with the distance travelled (PSettleDistance; [40]), but decreases with the number of adult foxes already in there (CrowdingFactor):

PSettle = PSettleDistance(Step) * CrowdingFactor(NumAdultFoxes)

PSettleDistance(Step) = (15% + (1-0.15) * Step/60)



**Figure 2:**
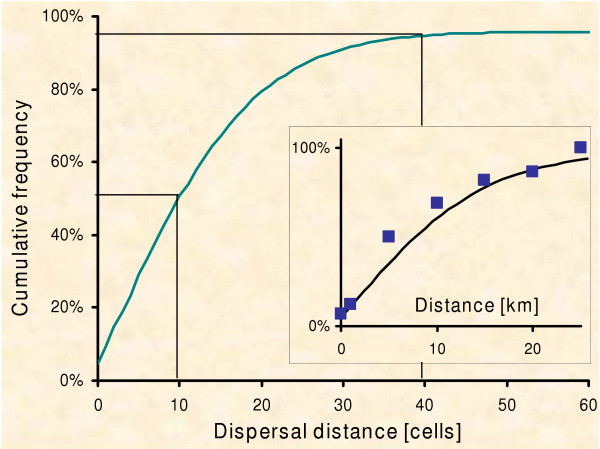
**Dispersal distances**. The cumulative distribution of dispersal distances as a result of the model algorithm. 51% move at most 10 cells, whilst only 3.5% disperse farther than 40 cells (indicated by vertical lines). The insert shows the dispersal kernel of the model together with field data observed by Jensen [43]. For this graph, the metrics of the cells are scaled as 0.8*0.8 sq. km.

The dispersal of one individual is limited to 100 steps, i.e. a maximum of 100 fox group home-ranges will be passed [43,70]. During each step we assume a mortality of 2% (adjusted to 22% dispersed foxes found dead by [69]). The emerging frequency distribution of dispersal distances is shown in Figure 2.

### Rabies transmission

Each fox has a disease state (susceptible, infected, infectious, or immune). The state is updated according to weekly time-steps. If infection was introduced in a cell by neighbourhood contact one adult fox is randomly selected. If this fox is not susceptible but "immune", nothing happens, otherwise its state changes from "susceptible" to "infected". The "infected" fox gets infectious after a negative exponential distribution with a minimum of 2 weeks and an effective mean of 3.5 weeks [71-74]. During the following infectious period of 1 week, a fox can transmit the disease [41,75]. It is assumed that infected cubs will die of rabies, but can only transmit the infection if their incubation period ends after the dispersal [15].

#### Local Contacts

An infectious fox passes the infection on to all other susceptible foxes within the cell [15,21,41].

#### Neighbourhood Contacts

If there is at least one infected fox in a cell, then the 8 neighbouring cells have a probability of 14% of getting infected [36,76], i.e. approach of Infection Communities [39] or 'group infection rate' in [41].

#### Mating Contacts (additionally in January and February)

If there is an infected fox in a cell, any neighbouring cell within a distance of up to 3 cells will be infected with a probability of 0.14^1^, 0.14^2 ^and 0.14^3 ^respectively [36,41,76,77].

#### Dispersal Contacts

There are hardly any infections during dispersal [15,53,67]. But juvenile foxes dispersing in their incubation period will cause standard transmission after settlement [15,78].

### Distribution of baits

#### Regional

Standard vaccination protocol on the regional scale comprises biannual campaigns with 18–20 baits distributed per sq km [7,10,11,13,17,79]. Accordingly, two vaccination events are performed in the model: one in the first week of April and one in the second week of September.

#### Local

**Figure 3:**
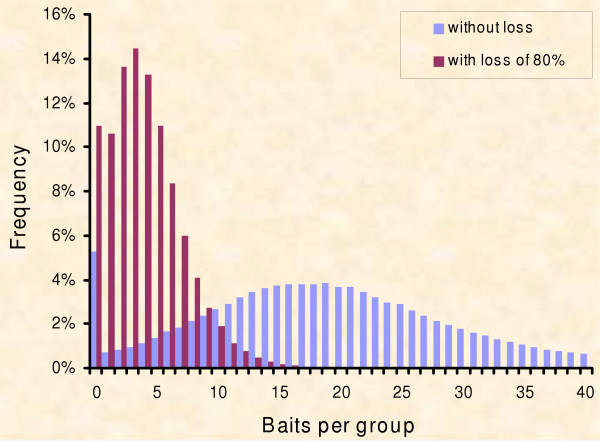
**Distribution of baits**. The frequency distribution of the number of baits received per fox group according to [82]. The grid model draws from this distribution and accounts for each bait a probability of 80% of being lost (e.g. to competitor animals) before assigning explicit baits randomly to individuals of a group.

**Figure 4:**
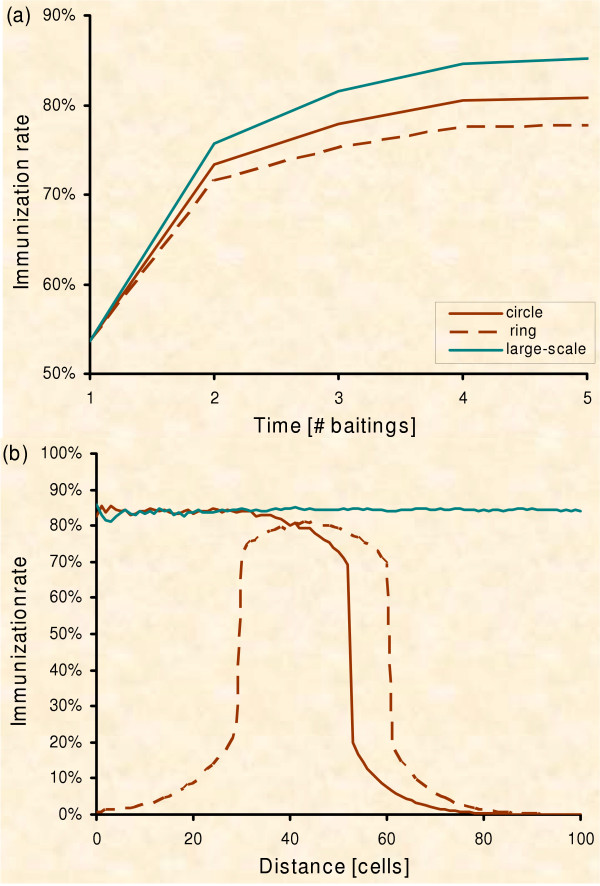
**Immunisation**. Immunisation level found in the fox population of the model (Circle – circle strategy; Ring – ring strategy [PVacArea = 10,800]; Large-scale – vaccination of the whole region). Biannual vaccination is always performed with 50% starting in autumn and 50% in spring. (a) Development of the immunisation level in the vaccinated area over time (100 repetitions): For the large-scale vaccination, both immunisation rate per campaign and final level of population immunity correspond to field data estimated during past control programs [16,87,91-93,104]. Dispersing non-immunised foxes lower the average immunisation level in the circular and ring-shaped vaccination areas. (b) The immunisation level after 3 vaccination campaigns by distance to the centre of the control area (100 repetitions): The immunisation level is lowered at the borders of the vaccinated area.

Grid cells represent the spatial equivalent of home-ranges of fox families, [38] which do not have equal area size [80] and hence will not receive an equal number of baits [81,82]. We approximate this non-equal assignment of bait pieces to spatial fox group home-ranges by simulating the distribution of effective bait numbers on the ground found for standard aerial delivery (Fig. 3) [82]. The baits randomly drawn to fall into a fox group home-range are assumed to be lost with 80% probability according to empirical findings, i.e. baits lost to competitors [51,83-88], baits unfound or only partly consumed [17,88-90]. The baits remaining in a particular cell are distributed randomly to the respective individuals, independent of their state. The "susceptible" foxes permanently turn towards "immune" two weeks after receiving at least one piece of bait [73,90,91]. With these rules an immunisation level of 70–80% emerges after 2 campaigns (Fig. 4) as empirically documented by vaccination campaigns in the field with 18–20 baits per sq. km [16,88,92,93].

### Emergency vaccination

#### Rabies detection

The mid cell of the grid receives an external infection during a randomly chosen week of the year. Subsequently, any rabies case will be detected with a probability of 2% [16,24,93,94]. Rabid juvenile foxes will be detected only from August onwards [15].

#### First vaccination campaign

If one infected fox is detected, we assume a preparation time of 2 months until the first vaccination campaign is scheduled (Vos, pers. comm.). Further campaigns are performed according to the standard protocol: autumn and spring [7,17,79] – with the only exception being that the second campaign will not be performed less than two months after the initial baiting.

### Spatial design – ring vs. circle

The relative assessment of the competing spatial designs is based on regular edges. Using the Moore neighbourhood, adjacent and diagonal cells are assumed to have equally scaled distances. The modelled emergency area is always centred on the first detected rabies case ignoring other "infected" cells on the grid.

The parameter vaccination area (PVacArea) corresponds to the maximum amount of cells that could be treated immediately after detection. PVacArea is set to be 6,400, 10,800 or 16,000 cells. The values are selected to provide useful width of the ring area (i.e. 20 km, 30 km or 40 km wide ring areas respectively). The area could be calculated into a necessary amount of baits after scaling the mean area of fox group home-ranges. For instance, in rural Europe fox group density of ~1 per sq. km is agreed [20,51] which fixes the mean area of the cells in the model at 1 sq. km Thus, the amount of baits per campaign used in the three scenarios is roughly: 128,000, 216,000 or 320,000 respectively when applying 20 baits per sq. km.

#### Circle strategy

The vaccinated area is compact around the detected outbreak and implemented in the model as a solid square. According to PVacArea, the region is 80*80, 104*104 or 126*126 cells respectively.

#### Ring strategy

**Figure 5:**
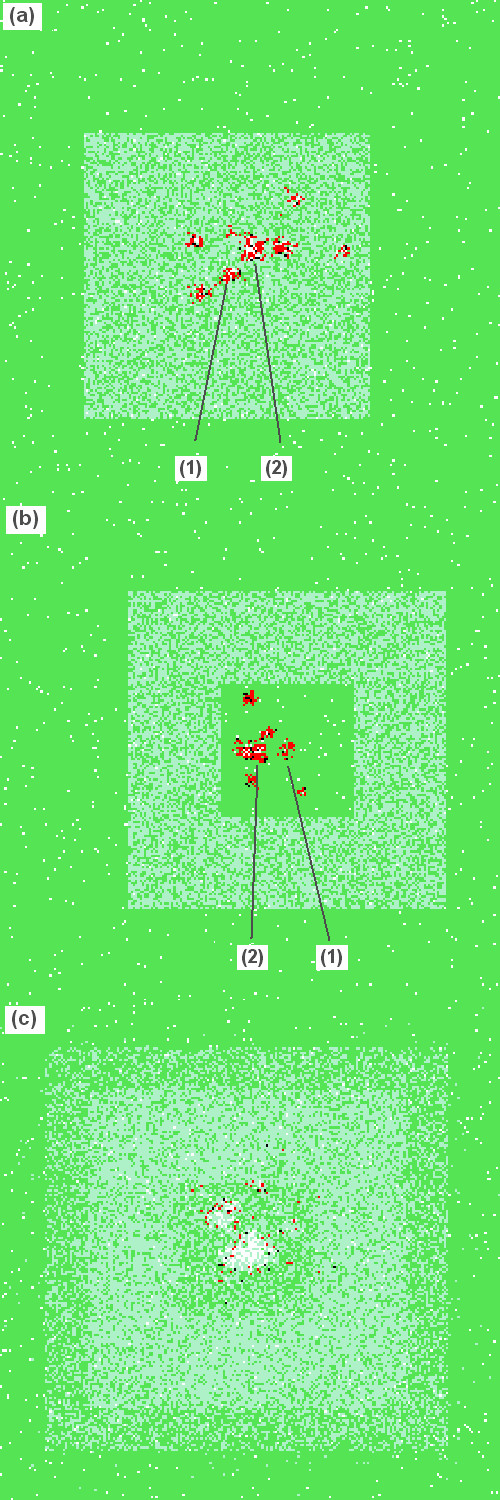
**Examples of simulation runs**. Example simulation run visualised after an infected fox was detected and the first vaccination was applied (PVacArea = 10,800; (1) – first detection of rabies set as centre of the control area, (2) – first infection of rabies). (a) Experiment 1: circle strategy. (b) Experiment 1: ring strategy. (c) Experiment 2: Ring strategy when PVacArea has been doubled. White – empty group, green – group of "susceptible" foxes, light blue – group with at least one "immune" fox, red – group with at least one "infected" fox, black – group with at least one "infectious" fox. If foxes at different states are within one cell, only the last of the list is shown.

60*60 cells remain without baits. This inner part should compensate for an annual spread of rabies of 30 km [27,41]. Around the interior, a ring of cells is assumed to be treated with baits. The treated area is determined by PVacArea and corresponds to a width of 20, 30 or 40 cells respectively [27]. The surrounded area (i.e. not baited + baited) thus covers: 10,000, 14,400 and 19,600 cells respectively. Figure 5(a) and 5(b) show screen shots of the simulations with these strategies treating an area of equal size.

### Simulation experiments

Simulation experiments are performed on a grid of 256 × 256 which totals 65,536 cells. We ran each simulation scenario 10,000 times to cover stochastic effects.

#### Experiment 1 – Containment of the epidemic with fixed resources

We assess which strategy performs better at confining the epidemic inside the control area over the short and long term. The frequency of infected foxes outside the control area provides the quantitative measure. The 3 sizes of vaccinated areas (PVacArea) remain constant throughout a simulation run.

#### Experiment 2 – Search for the optimal switch point from ring to circle strategy with increasing resources

The aim is to identify the strategy or a mixture of strategies which performs best in final eradication of the epidemic. The inner part of the ring has to be baited in the end to achieve eradication. Thus resource limitation is assumed to be eased at some point in time, and the baited area (PVacArea) is doubled afterwards. The resource extension is assumed with a lag of either 1 or up to 5 vaccination campaigns. Again, initial vaccination areas (PVacArea) will have 3 different sizes but they are doubled after the time lag. Technically, the following spatial configuration is applied in this experiment: Either we already start baiting the circle whose surface gets doubled after the time lag. Or, we start baiting the ring of the same size and after the time lag we continue baiting the circle of doubled surface, which of course contains the ring. Hence, the final treated area is always a solid square of 112*112, 146*146 or 178*178 cells respectively. Figure 5(c) shows an example of the configuration.

### Model 'robustness'

**Figure 6:**
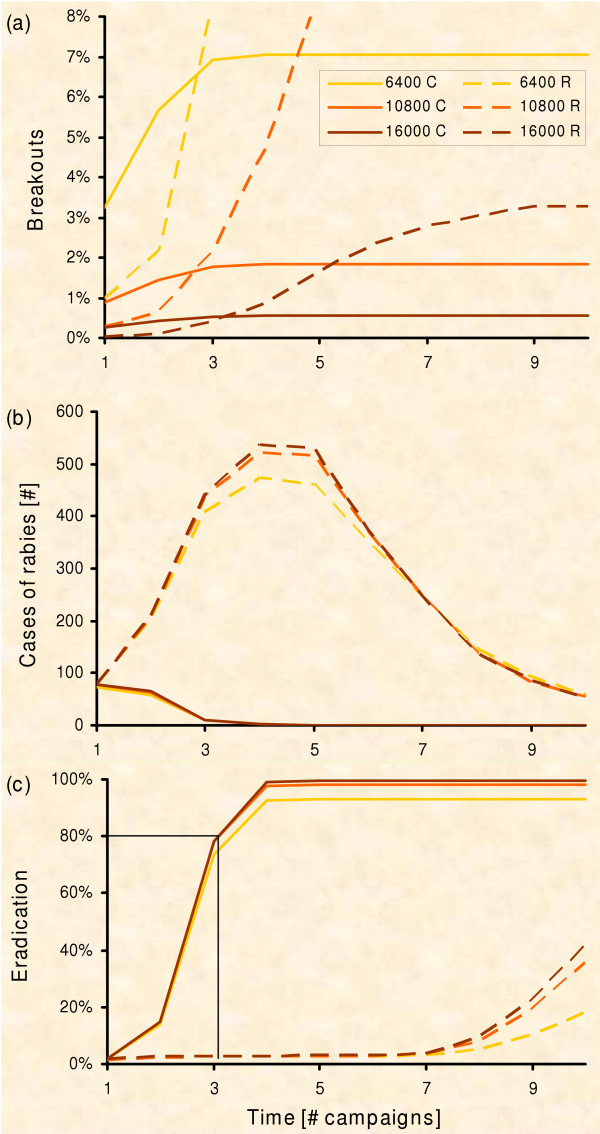
**Emergency vaccination with fixed resources**. Emergency vaccination with fixed resources (R – Ring strategy, C – Circle strategy; PVacArea = 6,400, 10,800, and 16,000; 10,000 repetitions). (a) Risk of a rabies breakout of the control area with respect to the number of vaccination campaigns performed: Initially there are fewer breakouts for the ring strategy, but in the long run, the circle strategy always performs better. (b) Average number of "infected" foxes between consecutive vaccination campaigns. Only simulation runs with rabies inside the control area are considered: As the inner part of the ring is not vaccinated, the epidemic can develop inside. (c) Chance of eradication with respect to the number of vaccination campaigns performed. With circle strategy rabies was eradicated in 80% of the repetitions after three vaccination campaigns (vertical line) but never with ring strategy.

We followed the pattern-orientated approach [49,95-98] for validation of the experimental results and qualitative debugging of the model logic [99,100]. Hence, we compared population parameters as re-read from the model to empirical data. The model successfully reproduces the fox ecology (e.g. fox densities during the year from around 1.5 to 3 foxes per sq. km [44,68,77,101], the dispersal distances (Fig. 2), the spread of rabies (Fig. 5b) [102,103], the development of immunisation level (Fig. 4a) [16,87,91-93,104] and the time period up to local eradication of an epidemic (Fig. 6c) [16,91,105]).

When parameters of the model were altered, only the quantitative results changed, but neither the qualitative results nor the conclusions did. But there is one noteworthy difference between large-scale and local vaccination concerning immunisation level. In emergency control the relatively small baited areas are surrounded by a susceptible neighbourhood and thus non-immunized foxes will regularly disperse into the baited region and vice versa. Indeed, the immunisation level maintained by the circle or ring strategy was measured lower than for the large-scale application (Fig. 4a), in particular at the edges of the control areas (Fig. 4b). Nevertheless, the resulting immunisation in the model was still sufficient to eradicate rabies locally, which is in agreement with recent findings about potential over-baiting during the past control programs in Europe [15,50,82,106,107].

## Results

### Experiment 1: Containing with fixed resources

In all scenarios we found noteworthy frequency of rabies infections spreading beyond the vaccinated area (Fig. 6a). For the medium amount of resources, about 1% of all simulation runs ended up with breakouts after 2 years. Independent of the amount of applied resources in the long run, the ring strategy performs worse than the circle strategy. In the ring strategy the number of rabies cases rises quickly (Fig. 6b) and the epidemic is not eradicated. On the other hand, by distributing the same resources in the circle strategy, the epidemic often gets eradicated (Fig. 6c). Only for the first two vaccination campaigns the ring strategy performs better in containing rabies.

**Figure 7:**
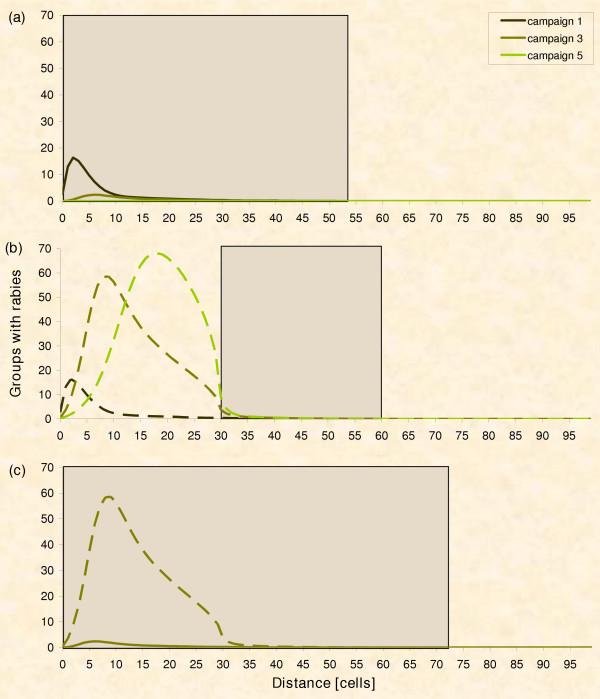
**Spatio-temporal spread of rabies**. The series show the frequency of infected fox groups at increasing distances from the centre of the vaccination area (average of 40,000 repetitions; solid line- circle strategy, hashed line – ring strategy; PVacArea = 10,800). The diagrams depict the frequency distribution after consecutive vaccination campaigns: black – after one; olive – after 3; green – after 5 campaigns respectively. The shaded areas indicate the extent of the vaccinated areas. (a) Circle strategy: The outbreak is soon suppressed. (b) Ring strategy: Rabies can devolve inside the non-vaccinated part. (c) Mixed strategies – Comparison of the epidemic situation just before control area is doubled (i.e. time lag = 3; hashed line = former ring strategy; solid line former circle strategy): There are more cases of rabies inside the final control area (i.e. shaded) when starting from the ring strategy, as compared to the former circle.

**Figure 8:**
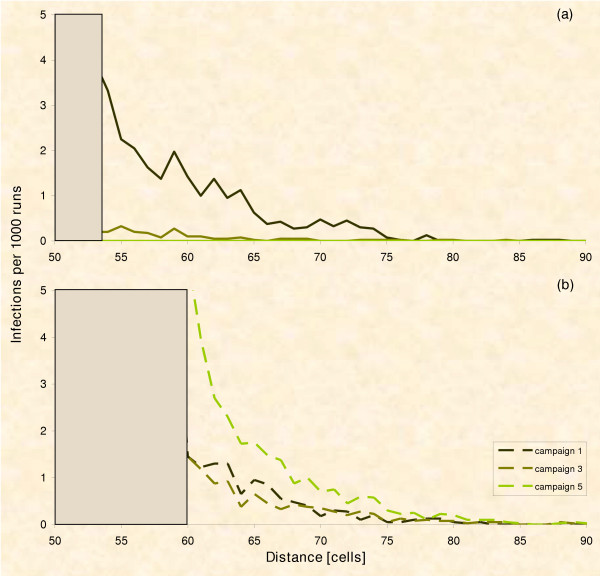
**Infections beyond control area**. Infections found beyond the control area's outer border (legend see Fig. 7, but notice that the x-axis was cut below 50 cells and the y-axis zoomed in because of the small numbers of recorded outbreaks). Only infections caused by foxes out of the control area are considered. (a) Circle strategy: Some cases might escape the smaller control area at the beginning. (b) Ring strategy: Fewer cases can escape initially, but the number of breakouts rises with time.

We detail the spatio-temporal dynamics of the simulated epidemic (Fig. 7 &8) to understand why the sole ring strategy performs badly. The strategy is characterised by an increasing risk of breakouts over time. Early breakouts are seldom due to the distant outer border of the control area (Fig. 7b &8b; black line). But only the ring itself is treated with baits and the epidemic can spread out within the non-vaccinated inner part (Fig. 7b). The growing number of infections close to the baited area challenges the ring (Fig. 7b; olive line) and, eventually, infections beyond the outer border of the ring rise with time (Fig. 8b; compare black and olive line). By contrast, the risk of breakouts associated with the circle strategy decreases with time. Soon after the outbreak, infections occur outside the circular control area, which would still be inside the control area under the respective ring strategy (comp. Fig. 8a &8b). However, the probability of eradication increases with time i.e. the number of vaccination campaigns (Fig. 6c), and thus the risk of still having an epidemic which could breakout diminishes (Fig. 8a; compare black and olive line).

**Figure 9:**
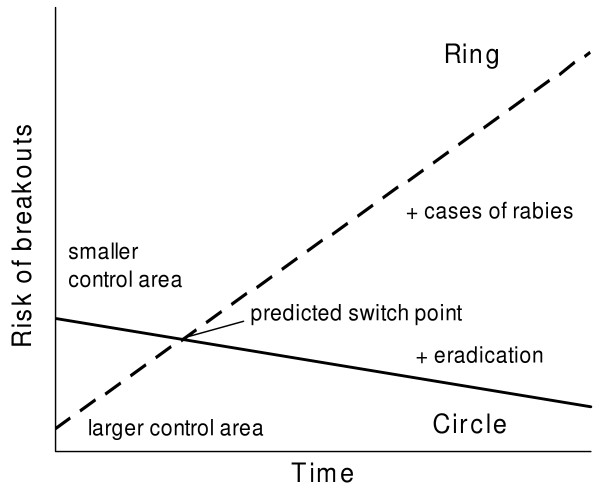
**Qualitative evaluation of risk of breakout**. The conceptual scheme depicts the risk of rabies breakout over time. Initially, the risk of rabies breakout is higher for the circle design compared to the ring design as the outer border of the vaccinated area is closer to the location of the detected rabies cases. The risk decreases as rabies ceases with repeated control. With the ring design, rabies can develop freely inside and the risk of breakout increases with time. Ring vaccination has to be stopped and eradication of the epidemic started no later than the switch point.

**Figure 10:**
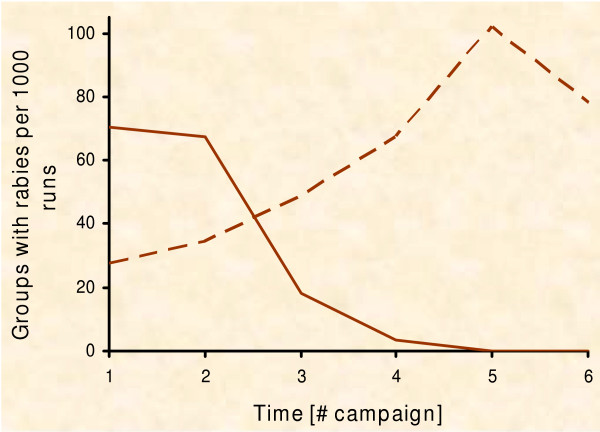
**Temporal risk analysis**. Frequency of infected fox groups beyond the border of the control area after repeated vaccination campaigns calculated from Fig. 8: Whereas the risk of infections decreases with the circle strategy, the risk increases with the ring strategy. The cross point is around the third vaccination campaign.

Figure 9 illustrates, qualitatively, the risk of breakouts over time. From this risk analysis we expect a crossover point before which the ring strategy has a lower risk and after which the circle strategy has the lower risk of breakouts. To check the prediction, we re-analyse data of Figure 8. We directly equate the risk of breakouts to the number of infections beyond the control area that are actually caused by foxes leaving the control area, and secondary infections are ignored (Fig. 10). Indeed, initially fewer infections are found beyond the outer border of the control area of the ring and later beyond that of the circle. Therefore, we attempt to profit from the initial advantage of ring vaccination by mixing strategies, i.e. starting control with ring vaccination (left down in Fig. 9) and later switching to the circle strategy (right down branch in Fig. 9).

### Experiment 2: Eradication with increasing resources

According to Figure 10 we expect at least one mixed strategy (switching from ring to circle after k baitings) to perform better than the continuous circle approach. Following this idea, we conducted experiment 2: The initial strategy is changed after k vaccination campaigns towards a circle application. In practice, the change could commence when resource limitations are overridden. Thus resources are doubled after the switch and the final circular control areas are IDENTICAL for all mixed strategies, i.e. ring-circle and circle-circle.

**Figure 11:**
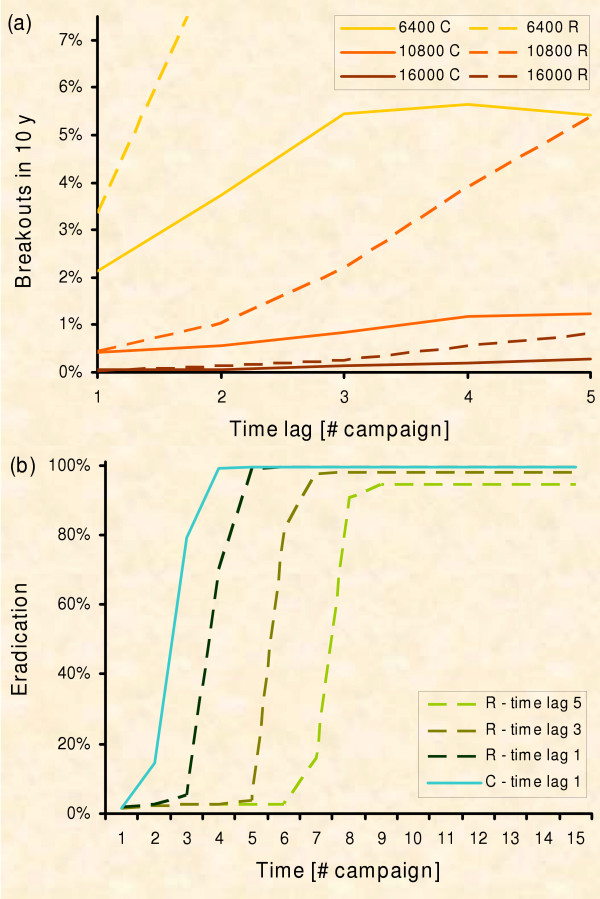
**Emergency vaccination with increasing resources**. The vaccinated area (PVacArea) is doubled with a time lag of 1 up to 5 vaccination campaigns (R – Ring strategy, C – Circle strategy; PVacArea = 6,400, 10,800, and 16,000; 10,000 repetitions). (a) Risk of rabies breakout from the control area with respect to the time lag when the vaccinated area was doubled: We cannot find the predicted switch point (see text); the circle strategy still performs better. (b) Chance of eradication with respect to the time lag when vaccinated area was doubled: In contrast to experiment 1 there is eradication now in the ring strategy; however the control success is shifted by the time lag.

Surprisingly, the strategy which immediately starts with a circle is still favourable (Fig. 11a). Indeed, the mixed strategy results in more breakouts and less eradication. Eradication also takes longer when using the mixed strategy as compared to the circle strategy (Fig. 11b), because the time lag before vaccination starts in the inner part of the ring is simply added to the time until eradication.

## Discussion

Expert knowledge and biological data about the host, the epidemiology of the infection or even the efficacy of management measures are quite often vague in the sense that they are never measured, they are examined with highly differing results or they are even difficult to sample precisely [52,108]. Nevertheless, it is necessary to overcome these uncertainties because management decisions have to be made [7]. Usually, modelling studies tend to select one particular configuration and substantiate the parameter choice with the help of logical arguments. We suggest an approach that is oriented to robust conclusions for practical management in terms of different or even antagonistic model assumptions. There are initial studies in the literature [109,110] which opt for the development of a more general methodology. The approach parallels standard techniques of model validation or sensitivity analysis. But we are no longer troubleshooting at the level of particular values which the model acquires in relation to slight changes in assumed parameters. We are only interested in changes at the level of conclusions made for management application, i.e. whether there are hypothetical scenarios which could falsify the management decision just derived from the model results. That is how practical management often performs [15]. Thus, while targeting useful support for these decisions, we have already covered the need for 'robustness' during the shaping of our management proposal.

We compared two spatial strategies of local emergency vaccination for controlling a rabies outbreak. One refers to the immediate control of infection within a smaller treated area. The alternative was theorised to overcome the drawback of a spatially limited strategy by providing equal resources in a ring around the affected area which contains the infection at the cost of more cases in the centre.

Simulation of the two spatial strategies revealed the true dynamics of the models. The ring strategy in general does not outperform the circle strategy. The predicted advantage of the ring strategy can only be found in the short term (Fig. 6a). Therefore, we determined mixed strategies and searched for the most rewarding point in time for switching between the ring strategy, which was better in the short term, and the circle strategy, which was better in the long term (Fig. 10).

Why aren't we able to identify the switch point as suggested by the risk analysis? Figure 10 actually proposes a switch around the third campaign. However, no matter which point we chose for switching from ring to circle in experiment 2, we did not find a clear advantage at all for the mixed strategy. There is only one plausible reason for this disagreement between the mind and the simulation model. The switch between the two strategies comprises a time lag until the protection level is reached inside the ring, i.e. until the inner part of the former ring actually acts like a circle. Our qualitative risk assessment (Fig. 9) assumes a perfect change between strategies. In practice however, we have to consider the temporal delay of building up population immunity which was shown to be at least 2 vaccination campaigns [16,87,91-93,104] in noteworthy agreement with our simulations (Fig. 4a). Thus the change in strategy has to take place two baitings in advance of the theoretical suggestion in order to profit with the mixed strategy. But the ideal switch point found in the simulation is around the 3^rd ^baiting campaign (Fig. 10). After subtracting the building up time of 2 vaccination campaigns in practice we need to change at baiting 1, i.e. we must apply the circle strategy from the very beginning.

Consequently, in the field we cannot benefit from the alternative baiting scheme and hence are forced to focus contingency planning on a compact control area around the detected outbreak. After testing the respective models, we can reject a-priori any pure vaccination field trial that attempts to distribute vaccine baits with a ring-like strategy.

Our findings are not contradicted by the successful application of cordon-sanitaire vaccination at borders of large-scale vaccination areas in the field [17,27,111-115]. In fact, the basic difference between a ring-like vaccination around a new outbreak and the cordon-sanitaire is the aim of control: In the outbreak situation we do not need to accept the rabies inside the ring, but in the second, the border situation, we have to. Rabies persists "on the other side" of the cordon, i.e. if neighbouring countries have not (successfully) combated the disease. Although the ring-like emergency vaccination does provide some protection for the surrounding area in the same manner in which the cordon-sanitaire vaccination does, in the emergency situation we aim for ultimate eradication, and in order to achieve this, our results clearly require the treatment of a compact circle-like control area.

The only threat for success of control is the migration of infected foxes from the limited area under treatment into the non-vaccinated surroundings. We cannot limit the distance infected foxes disperse, but we can reduce the number of them by means of the control itself. It is the circle strategy in which the number of rabies infections is lowered right from the beginning.

We present only 3 widths of the ring (Table 2). This is because ring width below 20 km cannot be expected to be protective [17]. Even though we analysed ring dimensions of 50 km and more in accordance with EU recommendations [17], there is no need to present these results. The difference between ring and circle strategy is less pronounced compared to that of the 40 km ring (Fig. 11a). Indeed, wider rings do provide decreasing gain in the outer radius due to the non-linear relationship between radius and area (Table 2, line 4). When the inner non-vaccinated part is reduced, there is no useful gain left, thus circle and ring become equivalent (Table 2, line 5). On the other hand, an extended inner part reduces the protective ability of the ring and simultaneously triples the non-vaccinated area (Table 2, line 6).

**Table 2:** Geometry of circle vs. ring. Gain in the maximum distance from the point of outbreak by using a ring instead of a circle. Presented scenarios with input parameters framed. Bold – respective calculations for scenarios not presented. Smaller inner non-vaccinated area reduces the gain, whereas a large inner radius reduces the thickness (i.e. protective ability) of the ring.

**Vaccinated area [km^2^]**	**ring thickness [km]**	**ring – inner radius [km]**	**ring – outer radius [km]**	**circle – outer radius [km]**	**gain [km]**
6400	20	30	50	40	10
10800	30	30	60	52	8
16000	40	30	70	63	7
***22000***	***50***	***30***	***80***	***74***	***6***
***10800***	***43***	***10***	***53***	***52***	***1***
***10800***	***22***	***50***	***72***	***52***	***20***

If the ring design strategically loses, i.e. in eradicating rabies, are there other benefits which outweigh the disadvantage? There are two other potential benefits to consider: economy and public health. With modern aerial bait disposal controlled by the GPS, logistic costs do not differ substantially between the two spatial designs (Mürke, pers. comm.). The cost is mainly linked to the total number of baits needed for the program. However, we demonstrated that in the ring design the time lag until the spared inner part is vaccinated directly adds to the time until eradication (Fig. 11b). This increases the cost of the ring design as compared to the earlier eradication in the circle design.

The remaining public health issue is the principal objective [7,71,116]. Eradication of the disease is the only method for achieving this objective [93]. However, within the inner part of the ring the epidemic roams freely and thus imposes a risk to humans and livestock which makes it less competitive than the compact circle approach. Additionally, eradication takes longer and thus the threat to public health is prolonged. Consequently, we can rule out the ring as a non-viable approach in terms of eradication, economy and public health.

In all respects we concluded that the circle performs better. But we still have to deal with possible early breakouts. Whilst zero risk strategies perhaps represent political demand, they are probably neither possible nor the most cost-effective approach. However, additional measures could be applied for an improvement of the performance of the circle strategy and will be considered in the ongoing analysis: Firstly, better monitoring programs could lower the time until detection of an outbreak, which consequently leads to an earlier eradication. Secondly, it is not clear whether immediate vaccination with a risk of imperfect placement performs better than waiting with the first vaccination until a monitoring program has provided a better understanding of the spatial extent of the outbreak. Thirdly, circles baited with spatially varying density of baits could provide both, the required fast suppression of the epidemic and the largest possible control area (see [31] for a combined simulation of poisoning and ring vaccination). And finally, follow-up programs can be designed. Indeed, the circle strategy with a control area of 10.800 sq. km has already provided a very low likelihood of breakouts. Thus, the strategic approach could extend the initial circular area to a non-circular control area (but still vaccinated on a regular basis), according to detected breakouts. Raised awareness after the reintroduction of rabies and particular border surveillance around the baited area, would guarantee fast detection of breakouts. Thus, we recognise the need for a more detailed cost-benefit, which explores the cost of extensions of the control area, versus the benefit of reducing the amount of resources applied to the initial hazard area.

## Conclusion

If vaccination is the only approved measure for fighting a rabies outbreak within a completely susceptible fox population, then the only feasible contingency plan is to vaccinate a compact area centred on the epidemic. The ring strategy which leaves an inner part non-vaccinated must be ruled out in all concerns: strategically, since it under-performs in eradication levels, economically, since eradication takes longer and public health, since it allows more cases of rabies.

Yet even in the circle strategy, there remains some risk of early breakouts of rabies from the control area. Thus, further studies should concentrate on optimizing the emergency strategy concerning timing and benefits of additional monitoring programs. Furthermore, a detailed cost-benefit analysis of potential strategic alternatives is needed in order to improve the outcome of a contingency plan.

## Competing interests

The author(s) declare that they have no competing interests.

## Authors' contributions

DE and HHT developed the model, and drafted the manuscript. DE implemented the code, and performed the simulation experiments. HHT, TS and TM developed the alternative strategy. TM provided the background of practical rabies management. All read and approved the final manuscript.

## Pre-publication history

The pre-publication history for this paper can be accessed here:


